# Visualization and Analysis Model of Industrial Economy Status and Development Based on Knowledge Graph and Deep Neural Network

**DOI:** 10.1155/2022/7008093

**Published:** 2022-04-28

**Authors:** Jing Quan

**Affiliations:** Xi'an Peihua University, Xi'an, Shannxi 710125, China

## Abstract

This paper adopts knowledge mapping combined with a deep neural network algorithm to conduct in-depth research and analysis on the current situation and development of the industrial economy and designs a visual analysis model of economic development based on knowledge mapping combined with a deep neural network algorithm. Cultivate the concept of coordinated development and legal system of the subject, improve the awareness of network security and integrity self-discipline of the subject, improve the level of network hardware equipment manufacturing, improve the level of network platform construction, build a network security technology prevention system, improve the repair system of network information alienation, set up a specialized agency setting for the coordinated development of network ecology and industrial economy, and increase the capital investment in network infrastructure and network information technology research and development. A framework of breadth and depth recommendation ranking based on a knowledge graph is proposed and implemented. This paper provides a visual analysis method to sort and classify multivariate data. The method first determines users' preferences through their interactive operations, calculates the weights of each attribute according to the users' preference model, then uses the obtained attribute weight sets to sort the whole data set, and finally completes the category classification according to the sorting results and the users' markings on some data. The visual display allows users to intuitively perform data sorting and classification operations and quickly understand the characteristics and category features of the data. The framework achieves modeling and integration of knowledge graph neighborhood information from breadth dimension and depth dimension to realize personalized recommendation sorting and improves the F1 metrics by 8.59%, 14.36%, and 15.22% on the public datasets Amazon-book, Yelp2018, and ILast-FM compared with the previous optimal model.

## 1. Introduction

In the era of Big Data, how to get value from the huge amount of data is a concern for enterprises and a hot research issue for researchers. Traditional data mining and data analysis methods can obtain information from the data, but how to make the information understandable to the users is another difficulty. Data visualization can present the hidden patterns and features of data in a graphical way, which enables users to understand the information quickly and intuitively in the data and improves people's cognitive and exploratory ability of data [1]. In the era of Big Data, visualization is no longer limited to scientific research and enterprise application fields, and interactive visual analysis of data and intelligent computing has become the common basis for major social needs, such as smart medical care, smart transportation, digital industry, and other aspects. In the face of the massive, heterogeneous, and multidimensional characteristics of Big Data, traditional visualization technology can no longer meet the analysis of these data, so the in-depth study of visualization technology is the need of the times, and the innovation of visualization technology will have a significant role in promoting the efficient use of Big Data resources [2]. With the information, acquisition methods, and means are constantly improving, for the increase of data volume, the way of data processing is usually simple statistics and query classification, much less direct analysis of the given data. As this category of data is based on the first national marine economic survey, the reported data have the characteristics of multiple categories and multiple periods, like the GM model, Laslie model, and other methodological models cannot make a comprehensive evaluation due to the limitations of their models [3]. In this case, there is a need for a more efficient and concise economic analysis solution and a more intuitive visualization solution to complement the analysis and prediction of the data than the conventional computer-based algorithms applied in the economic field, to efficiently solve the aforementioned problems.

Therefore, the research process of this paper mainly focuses on extracting valid data information from the surveyed data first in a semimanual and semiautomatic way. A visual analysis of coastal economic development based on a bottom-up grid clustering algorithm is proposed for the marine economic development of a region at the same time node, by inputting regional enterprise information and map area information in the algorithm to build a hierarchical queue, by calling the hierarchical queue to quickly plot the clustering results on the map and analyze the plotted results; for the problem of incomplete analysis due to the lack of time series [4]. In addition, we use the improved genetic algorithm to predict the marine economy industry and iteratively calculate the selection rate, crossover rate, variation rate, and other parameters to fit the original data through known data, and plot the final prediction results into the map and make predictions for the future development. Knowledge graph is mainly composed of data layer and schema layer. The schema layer itself is a tree-structured conceptual hierarchy relationship, including nodes and edges. Nodes represent an abstract concept, and edges represent relationships between concepts.

The goal of the recommendation system based on a knowledge graph is to build the association between people, things, and things with the help of the rich auxiliary information contained in the knowledge graph based on the historical user-item interaction data. To solve the data sparsity problem and cold start problem faced by the traditional recommendation system and improve the performance of the personalized recommendation system, the core problem is how to realize the recommendation scenario reasonably and effectively [5]. The core problem is how to realize the information integration of data and knowledge graphs reasonably and effectively. Currently, the mainstream recommendation algorithms based on knowledge graph focus on the recommendation ranking stage of personalized recommendation system, and the fusion of knowledge graph and recommendation system can be realized simply and quickly by directly constructing the connection between knowledge graph and recommendation candidates and integrating the neighborhood auxiliary information along the path of knowledge graph network. However, the related research is still in the initial stage [6]. There is a heterogeneous representation problem between recommendation scene data and knowledge graph-structured network representation. The mainstream research focuses on the recommendation ranking stage, and there is a lack of research related to the combination with knowledge graph in the recommendation recall stage. The recommendation ranking stage ignores the construction of higher-order combinatorial features of different neighborhoods of the knowledge graph, which limits the performance of the recommendation system. The practical landing experience of recommendation systems based on knowledge graphs in the field of science and technology service resources is still insufficient [7].

## 2. Related Works

A comprehensive and in-depth review of parallel coordinate visualization techniques is presented from the perspectives of constructing geometric models of parallel coordinates and methods for creating visual representations of parallel coordinates. Conventional parallel coordinates can show the negative correlation between two attributes by different visual patterns. However, it is difficult to identify positive correlations in parallel coordinate systems. In addition, highlighting multivariate correlations in parallel coordinate systems is not supported [8]. When the data set is too large, the parallel coordinate and scatter plot matrices are difficult to represent, the spacing of the parallel coordinate axes shrinks when there are too many dimensions, and the matrix in the scatter plot shrinks when the amount of data is too large, which makes it impossible for the user to identify the relationships between the data [9]. This problem can be solved by using dimensionality reduction technology to spatially transform the multidimensional attribute data and project them into two or three dimensions to help users intuitively access the differences and characteristics of data objects in higher-dimensional space. The early web-based encyclopedia-oriented entity linking methods mainly use some manually designed rules to match entity mentions with candidate entities. These include textual overlap-based methods and web encyclopedia page feature-based methods [10]. The text overlap-based method measures the matching degree of entity linking results by designing certain metrics to measure the overlap between natural language text and the page description text of entities in the web encyclopedia. The methods based on the characteristics of web encyclopedia pages use page elements, such as bold, underline, external links, and information boxes, in web encyclopedia pages to design certain rules to measure the matching degree of entity link results. These methods are too simple and lack accuracy and flexibility [11]. In 2007, Vaishya et al. improved the traditional restricted Boltzmann machine (RBM) model by combining the collaborative filtering method with RBM for the first time and applied the model to movie recommendation, which effectively improved the accuracy of recommendation [12].

In this case, the conventional model prediction algorithm is very prone to inaccurate prediction due to the inability to consider spatial factors, time correlation, and other factors; the data collected from the first national marine economy is often presented through a single list to show, unable to intuitively see the development of the marine economy, more need a visual way to show [13]. The actual performance is mainly reflected in the hotter regions with the denser distribution of enterprises or the status quo of individual enterprise profits showing very high production value. Since the characteristics of the data vary at different scales, it seems to be a spatial pyramid-like clustering problem. Lizunkov et al. proposed AutoRec algorithm by combining self-encoder and collaborative filtering methods, which uses self-encoder to extract features of users or items and optimizes the model by minimizing reconstruction error [14]. Zhang proposed the collaborative recurrent self-encoder model CRAE, which uses recurrent neural network to model text sequences in both encoder and decoder to obtain the information conveyed by word sequences in text. sequences in the text [15].

It is difficult for a region to experience a sudden increase or a rapid decrease in development, and the trend of the year is often closely related to the previous year or even to previous years. Therefore, such a characteristic can be regarded as a kind of heritability, and the data characteristics of the year are likely to be the result of continuous-time co-weighting, and genetic algorithms often need to combine with the information of the previous time-series features to deduce the subsequent changes, which is very similar to the two situations, from this point of view, this is another genetic algorithm prediction problem [16]. To address the heterogeneous representation of recommendation scenarios and knowledge graphs, we first define and construct a compressed collaborative knowledge graph, based on which we propose a two-tower recall model based on the compressed collaborative knowledge graph and experimentally verify the effectiveness of the proposed method on public datasets. We focus on the limitations of knowledge graph information integration in existing knowledge graph-based recommendation ranking algorithms, propose a breadth and depth recommendation ranking framework based on the knowledge graph, integrate the knowledge graph auxiliary information from the breadth and depth dimensions, and experimentally verify the effectiveness of the proposed method on public data sets.

### 2.1. Knowledge Graph Fusion Deep Neural Network Algorithm Design

Google first proposed the Knowledge Graph to improve the search quality of search engines and thus the user's search experience. The essence of the knowledge graph is a semantic network that stores entities and inter-relationships between entities to describe the real-world things themselves and the connections between things. The knowledge graph is mainly composed of a data layer and a schema layer. In terms of data visualization, the main task of data processing is the transformation of data format, and the collected raw data must be converted into a data representation that is convenient for system processing. The schema layer itself is a tree-like structure of concept hierarchy relations, including nodes and edges, where a node represents an abstract concept and an edge represents the relationship between concepts. The data layer contains nodes and relationships, where a node is an instance or can be an attribute value and an edge is a relationship between entities.

Knowledge graphs can be divided into open-domain knowledge graphs and domain-specific knowledge graphs by application. Open-domain knowledge graphs contain many nodes and relationships and have a certain tolerance to the quality of knowledge extraction, so the accuracy is often difficult to judge. Domain-specific knowledge graphs require higher knowledge quality, have more complex knowledge structures, integrate domain data from multiple sources to expand the scale, and pay more attention to the accuracy of knowledge. Usually, the semantic information of entities is high-dimensional and sparse, so it is necessary to reduce the dimensionality of entities and relationships in the knowledge graph by knowledge representation and represent entity vectors in a low-dimensional dense space [17]. Knowledge representation learning is also called knowledge embedding, for example, embedding song information into the knowledge graph can be derived from the song vector.

**Figure 1:**
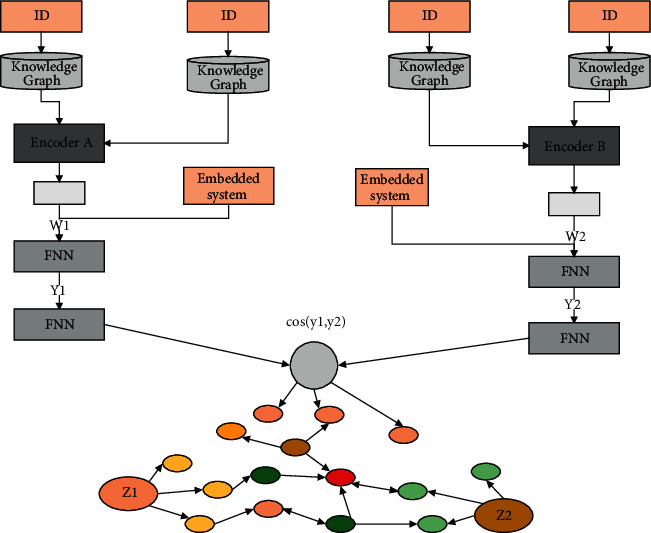
Overall architecture of the two-tower recall model for knowledge graphs.

The two-tower recall model of recommendation scene data and knowledge graph data. To address the heterogeneous representation problem between recommendation scene data and knowledge graph data, a compressed collaborative knowledge graph (CCKG) is defined and constructed to realize the integration of recommendation scene data and knowledge graph data in the space of implicit semantic representations. On this basis, a twin-tower recall model based on a compressed collaborative knowledge graph is proposed, which draws on the modeling idea of DSSM implicit semantic representation and integrates the knowledge graph neighborhood information through a transformer encoder to realize the mapping of users and items in recommendation scenarios into the same implicit semantic space, while using a compressed collaborative knowledge graph as a bridge to establish the features between users, items, and knowledge graphs. The interaction between user, item, and knowledge graph is established to obtain more accurate user interest preference features and finer-grained item attribute features to improve the accuracy of recommendation recall. The overall architecture of the twin-tower recall model based on the compressed collaborative knowledge graph is shown in Figure 1.

In the process of semantic information extraction of entity mention contexts, the method combines semantic dependency analysis techniques and graph convolutional neural network algorithms to model the semantic dependency features an entity mention context, which can fully extract the key information in entity mention contexts. In the process of mining semantic information of knowledge graph entities, the method uses graph attention networks to model structured information in the knowledge graph, which can selectively consider the graph topology features of entities and obtain a more targeted knowledge graph entity vector representation. The experimental results of the proposed model are verified to outperform the comparison methods on five publicly available data sets.

Graph data is a data type that represents data objects and relationships between data objects. The nodes in the graph represent specific data objects, and the edges in the graph represent relationships between data objects. In a graph, the information represented by a node includes the node's characteristics and the set of neighboring nodes that are related to the node. Graph data is widely used, and the knowledge graph studied in this chapter is one kind of graph data.

With the development of deep learning techniques, modeling graph data using deep neural networks has also gradually become a hot research topic. The existing neural network methods are extended to the field of graph data. A graph neural network is a deep learning method for modeling graph data, which can model the target node using its features and the set of neighboring nodes to obtain the vector representation of the target node *h*. The vector representation of the node is fed into an output function g to obtain the final output result. This process corresponds to the following equation:

(1) $$\begin{array}{c} h_{v}=f\left(X_{v},X_{\mathrm{cov}}\right)+g\left(h_{v},x_{nev}\right),\\ o_{i}=f\left(x\right)+g\left(h_{i},x_{i}\right), \end{array}$$

where *f* is called the transition function, *x* is the own feature of node *v*, *xv* is the relational feature of node *v*, *h*_*e*_ is the vector representation of node *v*'s neighbor nodes, and is the feature of node *v*'s neighbor nodes. The loss function of the graph neural network is defined as follows:

(2) $$\begin{array}{c} \mathrm{Loss}=\sum_{i=1}^{p}\left(t_{i}+o_{i}\right). \end{array}$$

In the entity-mentioned context encoding, the DPGAT method first encodes the overall context using a bidirectional long- and short-term memory network. Subsequently, the semantic dependency analysis is performed on the entity-mentioned context, and then the semantic dependency graph is constructed. Finally, a graphical convolutional neural network is used to encode the semantic dependency graph to obtain the encoding of semantic dependencies in the entity-mentioned context. Another key point of data processing is how to express data from different sources and types in a unified format to facilitate later data mapping, drawing, and analysis. The final encoding result of entity mention context is obtained by combining the encoding of the overall context and the encoding of semantic dependencies. In the candidate entity encoding, the DPGAT method uses the graph attention network to encode the candidate entities with the help of the set of neighboring entities of the candidate entities and assigns different attention weights to different neighboring entities.

Different aspects of an entity are considered, and respective relationship spaces are established for different relationships, and the entity vectors are mapped to the relationship space by a mapping matrix *M*_*l*_ when performing calculations on the entity, that is,

(3) $$\begin{array}{c} h=hM_{l},\\ t=tM_{l}. \end{array}$$

Using the output of the multiheaded attention layer as input, the higher-order information feature representation of the user or item integration neighborhood is extracted by a nonlinear transformation of the two fully connected layers, and the specific computational procedure is as follows:

(4) $$\begin{array}{c} FFN\left(x\right)=\frac{x\left(w_{0}-b_{0}\right)}{W_{1}}+b_{1}. \end{array}$$

In addition to this, layer normalization and residual summation are processed between the multiheaded attention layer and the feed-forward network layer, and between the feed-forward network layer and the final neighborhood information feature output, which are intended to speed up the gradient descent parameter learning process of the network and avoid problems such as gradient disappearance.

**Figure 2:**
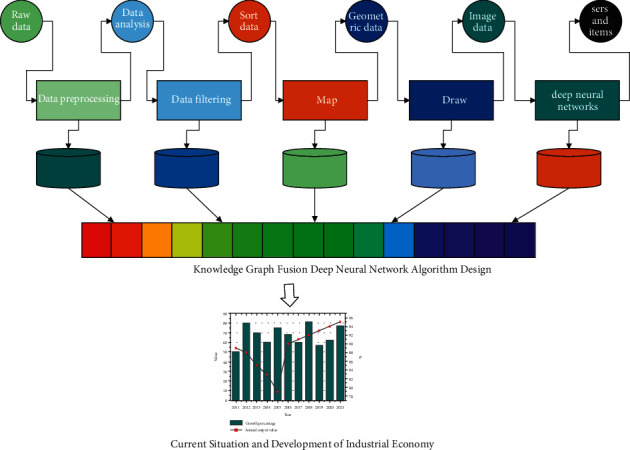
Visualization pipeline for scientific visualization.

The model maps user query and text separately into the same hidden semantic space employing dual deep neural networks and then realizes the representation and matching of the user query and text content. With its excellent ability to extract and characterize the hidden semantics, DSSM is widely used in the recommendation recall stage to match users and items, and its solution idea is to construct the corresponding hidden semantic feature representation vectors from the user side and the item side respectively, and to obtain the similarity ranking results of the candidate items by cosine similarity calculation based on the hidden semantic feature representation vectors of users and items to obtain personalized recommendations. A subset of candidate items is recalled, as shown in Figure 2.

If the latent semantic features of the user and the item are closer, the cosine similarity between the latent semantic feature vectors of the two is greater, and it can be considered that the user is more interested in the item. Data processing refers to steps such as data preprocessing and statistical analysis of raw data, to ensure the consistency, correctness, and integrity of the data. For data visualization, the main task of data processing is the transformation of the data format, where the collected raw data must be converted into a form of data representation that is easy to process by the system. Data are uncertain, as each data point is a quick capture of what happened at a given moment, so the representation of massive amounts of data is challenging, mainly in terms of the methods used to be able to faithfully maintain the content and characteristics of the data. Another focus of data processing is how to represent data of different sources and types in a uniform format for later data mapping, plotting, and analysis.

In this paper, based on the design idea of the aforementioned two-tower model, the neighborhood information feature vectors of users and items are obtained to achieve the final hidden semantic representation of users and items. Based on the model architecture of DSSM for hidden semantic representation learning, the initial embeddings of users and items are spliced with the corresponding neighborhood information feature vectors for the current recommendation recall query, which are used as the input of user side and item side, respectively, and the extraction of hidden semantic feature vectors is realized by a dual deep neural network:

(5) $$\begin{array}{c} E_{u}^{1}\geq \text{Concat}\left(E_{u},E_{i}^{1}\right). \end{array}$$

The overall computation process of the twin-tower recall model based on a compressed collaborative knowledge graph is introduced in detail. After obtaining the implicit semantic feature vectors of users and items, the recommendation recall matching needs to be realized by calculating the semantic similarity between them, and the common similarity calculation methods are cosine similarity, Euclidean distance, Hamiltonian distance, and so on. In this paper, we use cosine similarity to measure the implicit semantic similarity between users and items. For the current candidate prediction, if the implicit semantic features of users and items are closer, the greater the cosine similarity between their implicit semantic feature vectors, the higher the interest of users in the item can be considered:

(6) $$\begin{array}{c} R\left(u,i\right)=\gamma \frac{E_{u}^{1}E_{i}^{1}}{\text{cosine}\left(E_{u}^{1},E_{i}^{1}\right)}. \end{array}$$

### 2.2. Visual Analysis Model Design of the Current Situation and Development of Industrial Economy

A visualization schema is a generalization of the data presentation format. Common data visualization patterns include tag cloud, sequence analysis, network structure, and e-map. The visualization schema is generally selected based on the goals and requirements of the visualization. The first task of data visualization is to select a suitable visual coding, and which visual coding to be used is determined by the characteristics of the perception system, the properties of the data, and the task objectives. In the era of Big Data, the large volume of collected data and the dynamic nature of the data require visualization methods that can not only display statically but also efficiently and dynamically.

Convenient interactive visual operations can help people enhance their exploration of data and assist them in iterating through the data to get the desired results. Human-computer interaction has long been used in various fields, but interactive visual analytics for large amounts of data is still being explored [18]. The main challenges include the design of interaction methods, complex interaction processes, innovative interaction concepts, and the applicability and expandability of interaction methods.

**Figure 3:**
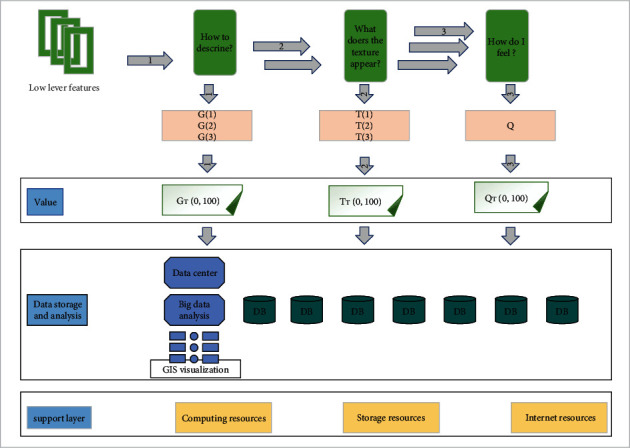
Visualization analysis model architecture.

The purpose of visualizing data is to allow users to quickly and intuitively discover the patterns implied in the data or gain knowledge from it. However, if the visualization is not well designed, it is likely to end up with graphics or images that are incomprehensible to the user and do not effectively convey the meaning of the data. In addition, without good interaction, users will not be able to analyze the data deeply enough to explore it in depth. Designing a good visualization method is the only way to improve the user's ability to perceive the data. Therefore, understanding the design principles of visualization and the functions of some components is the basis for designing an effective visual analysis, as shown in Figure 3.

The goal of coordinated development of network ecology and the industrial economy is not to reach the optimal state for both, nor to reach the optimal for one party, but to promote the development of each other as the most suitable goal the most suitable goal of industrial economy development is relative to the network ecological environment. The most suitable goal of industrial economic development is to improve the environment and conditions of people's network information activities and to realize the best goal of the network ecological environment. Take the lead in taking the network ecological environment protection as the foundation and core, follow the principle of protection priority, and promote development in the network ecological environment protection, and the two complement each other. On the basis of emphasizing the commonalities, taking into account the differences between the two, the ultimate goal is to realize the orderly development of the industrial economy and the network ecological environment. The coordinated development of industrial economy and network ecological environment is not based on the optimal industrial-economic goal or the optimal network ecological goal but on the specific network ecological environment and network information resource conditions, so that the development goal of an industrial economy and the most suitable network ecological environment construction goal can achieve organic unity.

Leaving the foundation of the network ecological environment, the pure industrial-economic goal is just a single-minded pursuit of industrial economic growth. Even if this goal can satisfy the greatest human desires, it destroys the foundation of industrial economic development, and such an optimal goal of industrial economic development is unsustainable. Likewise, the most appropriate goal of network ecology is related to the development of the industrial economy and is the best goal to meet the development of the industrial economy. Without the goal of industrial economic development, the pure goal of network ecology construction is of little significance to human society. The coordination of industrial economy and network ecology not only emphasizes the coordination of the development speed of industrial economy and network ecology environment but also emphasizes the rationality of the structure ratio between each element and system, such as emphasizing the reasonable allocation of network information resources. Through the setting of appropriate network ecology and industrial economy coordinated development goals to effectively regulate the industrial economy growth mode, and continuously improve the industrial economic growth speed and industrial economic development quality to achieve the benign development of industrial economy and network ecological environment.

**Figure 4:**
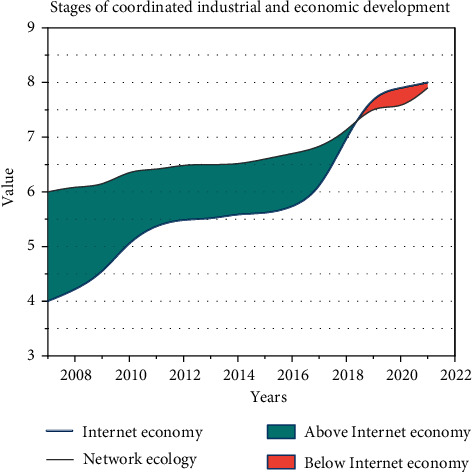
Stages of coordinated industrial and economic development.

The development of controllability simply means that we cannot simply pursue the brutal growth of the industrial economy, and we should pay enough attention to the network ecology and environmental protection in the process of industrial economic growth. In the coordinated development of network ecology and industrial economy, it is necessary to fully understand the inter-relationship between the two, follow the inherent rules, adopt technical means or institutional means, such as through macro-control, to regulate and control the process of change of network ecology or industrial economy, prevent blind development, and promote the coordinated development of network ecology and industrial economy. The coordinated development between network ecology and the industrial economy is more scientific and reasonable, as shown in Figure 4.

Dynamic nonequilibrium is to emphasize the difference between industrial economic development and network ecological environment, not to require unprincipled network ecological construction and industrial economic development must be balanced, but when the industrial economy has reached a certain level of development, the contradiction between industrial economy and network ecological environmental protection and exceeds the carrying capacity of network ecological environment, take the lead to take network ecological environmental protection as the basis and core, and follow the principle of protection firstly. The two complement each other by promoting development in the network ecological protection. The goal is to achieve an orderly development of the industrial economy and the network ecosystem while emphasizing the commonalities and considering the differences between the two.

Using user-item pairs as input, we retrieve finer-grained attribute features of items in the knowledge graph (i.e., the neighborhood of the knowledge graph), and initialize the embedding feature representations of users, items, and other entities through the embedding layer. In the breadth dimension, we combine the user, head entity, relationship, and tail entity to model the neighborhood triad feature representation, and use the triad compression interaction network to achieve the construction of finite higher-order combinatorial features among the triads of the knowledge graph; in the depth dimension, we use the user and relationship to model the neighborhood tail entity weights, and weight the sum to achieve multihop neighborhood information integration; in addition, we additionally introduce linear units to retain the shallow feature information.

## 3. Analysis of Results

### 3.1. Knowledge Graph Fusion Deep Neural Network Algorithm Performance Results

**Figure 5:**
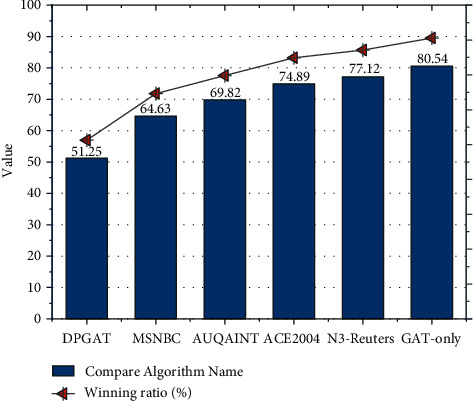
Entity link F1-score of DPGAT and comparison methods on MSNBC data set.

This chapter is designed to train DPGAT with other comparison algorithms on the AIDA-train data set, test them on the AIDA-A data set, and evaluate them on MSNBC, AUQAINT, ACE2004, and N3-Reuters. The experimental results are shown in Figure 5. From the experimental results, the DPGAT algorithm can improve the F1-score of the knowledge graph-oriented entity linking task in general compared with the comparison algorithms, which will be analyzed separately for different data sets in the following [19].

The F1-score of DPGAT and the comparison method on the data set MSNBC are shown in Figure 5. From the figure, it can be seen that: the F1-score of the DPGAT algorithm achieves a 2.2% to 29.3% improvement compared to the public benchmark comparison method; the F1-score of the DPGAT algorithm achieves a 0.7% improvement compared to the internal benchmark comparison method.

First, the F1-score of the Meta-EL algorithm proposed in this chapter is generally better than the public benchmark comparison methods on the five data sets, which validates the overall effectiveness of the DPGAT algorithm proposed in this chapter. Second, compared with the feature engineering-based methods DBpedia Spotlight, AGDISTIS, and DoSeR in the public benchmark, the F1-score of DPGAT on all five data sets is substantially improved, indicating that the automatic feature extraction method using deep neural networks outperforms the traditional feature engineering methods and can substantially improve the performance of entity linking methods. In the public benchmark, the F1-score of DPGAT method is better than that of CoGCN, which shows the effectiveness of this method. With the increase of *γ*, the precision and recall rate of the model increase accordingly. When *γ* is equal to 0.9, the two indicators reach the maximum value, after which *γ* increases, and the precision rate and recall rate decrease. DPGAT algorithm in combining semantic dependency analysis and graph attention networks. Finally, the F1-score of DPGAT outperforms the internal benchmark method GAT-only, which indicates the effectiveness and necessity of semantic dependencies in entity-mentioning contexts for entity linking, and shows that the DPGAT method can effectively extract the semantic dependencies in entity mentioning contexts using semantic dependency analysis techniques and accomplish the entity-linking task more accurately.

**Figure 6:**
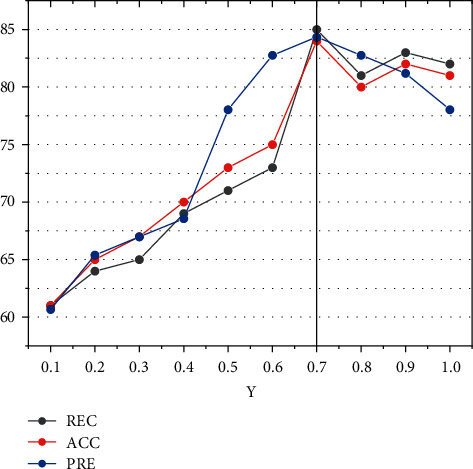
Performance impact of the algorithm model DRLRS.

The effect of the discount factor *γ* in reinforcement learning on the model recommendation performance is shown in Figure 6. The horizontal coordinates of these two plots are the values of *γ* in the range of [0.5, 0.6, 0.7, 0.8, 0.9, 1], and the vertical coordinates are the values of accuracy and recall. The experiment is conducted on the JData data set and KKBOX data set, and the impact of the discount factor on the recommendation performance of the model is measured by calculating the two metrics of accuracy and recall. From the experimental results, we can see that the accuracy and recall of the model increase as *γ* increases, and the three metrics reach their maximum values when *γ* equals 0.7, after which *γ* increases and the accuracy and recall decrease.

The discount factor is used in the Markov decision process to adjust the weighting of current payoffs to future payoffs. The closer the discount factor is to 0, the more emphasis the reinforcement learning model places on immediate current payoffs; the closer it is to 1, the more emphasis the model places on long-term future payoffs. A higher discount factor (between 0.9 and 1.0) makes the intelligence afraid of the future and unwilling to explore; a lower discount factor makes the intelligence dare to explore the environment (e.g., 0.9) and find the optimal path through trial and error; a too-low discount factor (e.g., 0.5) makes the intelligence unable to explore the environment because it does not receive future feedback. So, it cannot find the optimal path. For larger e-commerce data, many decision behaviors are generated in a day, so the reward value after multiple behaviors needs to be considered, and the value of the discount factor will be close to 1. However, too large a discount factor will cause the model to be difficult to converge.

**Figure 7:**
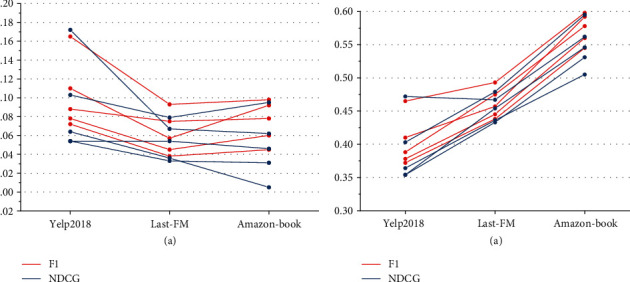
Performance under different breadth dimension neighborhood sampling numbers.

A deep neural network for building user states is constructed, which introduces user behavior sequences, models behavior sequences separately consider both the transition relationship between behaviors and the influence of current behavior on user preferences, mines user preferences in a fine-grained way, and obtain dependencies of long sequences with a self-attention mechanism to reduce information loss. The whole model is trained by deterministic policy gradient DDPG, as shown in Figure 7.

For different data sets, the performance of recommendation ranking results is compared by adjusting different depth dimension neighborhood sampling numbers, it can be found that similar to the breadth dimension, the performance of KGWD shows an increasing trend as the number of depth dimension neighborhood sampling *N* increases, which indicates that the more the number of depth dimension neighborhood sampling *N*. The more long-distance interest preferences of users can be found, but too-large depth *N* may cause overfitting, resulting in a decrease in recommendation performance, and also occupy too much memory and increase the computational burden. Whether to adopt a broad and shallow or narrow and deep information architecture model needs to be comprehensively considered according to the industry, user needs, and user professional skills.

It indicates that the graph structure-based model can better characterize items by capturing the higher-order connectivity of items and mining the complex relationships between items, thus improving the recommendation performance. DDPG-KNN, TPGR, and PGPR are all reinforcement learning-based recommendation models, and all three outperform the deep learning-based model in all three-evaluation metrics on both data sets. The performance of DRLK-RS improves compared to DRLRS, with a 1.91% improvement in accuracy and 2.76% improvement in recall on the JData data set. The performance of DRLK-RS improves compared to DRLRS, with 1.91% improvement in accuracy, 2.76% improvement in recall, and 1.52% improvement in normalized discount cumulative gain on the JData data set, and 1.7% improvement in accuracy, 2.84% improvement in recall, and 2.46% improvement in normalized discount cumulative gain on the KKBOX data set, indicating that adding knowledge graph can improve recommendation performance to some extent.

### 3.2. Validation Results of the Visual Analysis Model of the Current Situation and Development of the Industrial Economy

The original purpose of data visualization is to map a wide variety of multisource heterogeneous data sets into geometric elements through algorithmic design and then output graphical images to show the structure and insight into the laws in an intuitive, clear, and concise manner. Data is an asset, and data visualization is an important tool for revitalizing this asset [20]. The set graphical style for the data set is the same kind of homogeneous merging, the link graphical style for the related data set is the relationship mapping, and the part-whole graphical style for the complex data set is the surface of the point is the outstanding embodiment of simplification. Properly integrated use of related composite schemas in the process of visual representation design can provide intuitive insight into patterns, facilitate knowledge acquisition, and properly maximize the value of information.

**Figure 8:**
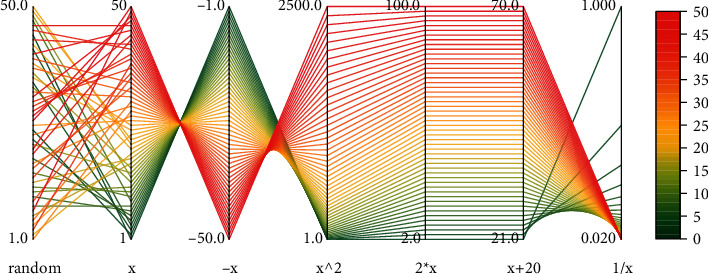
Parallel coordinate visualization mapping.

It is not only the correspondence between the part of data visual graphics and the whole but also the correspondence between the user's operation intention and the operation behavior when interacting with the complex data visual graphics; the correspondence between the operation behavior and the visual effect; the correspondence between the operation state and the system state perceived by the user through sight, sound, and touch; the correspondence between the perceived system state and the user's demand and expectation, as shown in Figure 8.

In the visualization of multidomain heterogeneous data, there is both the demand for the panoramic display of large-screen terminals and the limitation of local display of mobile terminals. The visualization of complex multisource data is difficult to be displayed in limited screen space, so it is especially important to make reasonable partitioning of the map to adapt to different display terminals. Atlas segmentation, display terminal segmentation, and collocation are all issues that need attention in visual design. In the project of visual expression of tea data in a certain place, in which I participated in the design, both the panoramic display of the large-screen terminal and the small screen display mapping were designed for the cell phone terminal.

Information architecture is one of the important features of multidomain heterogeneous data visualization, whose main task is to build an easy-to-perceive bridge between high-dimensional complex data and user perception, and this architecture is the carrier of abstract data visualization. Data visualization that can be presented on a single page often adopts a narrow and deep information architecture model, which takes up less screen space for users to explore the knowledge contained in the data independently. Multidomain heterogeneous data visualization is difficult to present all information on a single page and requires the design of information architecture, which combines multipage presentation with visualization to facilitate users' insight into data association and extraction. Whether to adopt a wide and shallow or narrow and deep information architecture model needs to be considered based on the industry, user needs, and user expertise.

**Figure 9:**
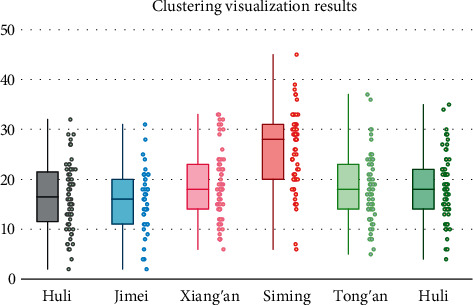
Clustering visualization results.

In the design practice of data visualization and other information systems, many designers have consciously or unconsciously become aware of this metaphor and use it, especially in the design of complex systems. Intuitive user interface metaphorical mapping makes the system more inclusive, and this mapping exists not only in the visual graphics but also in the human interaction actions with the visual system. At the same time, since imagery schemas and their metaphors are often activated by repeated events in life, they have become a kind of subconscious, which allows users to navigate information systems designed based on metaphorical mediation without invoking too many mental models and too high a learning cost, as shown in Figure 9.

Compared to model assessment visualization, clustering visualization tends to be more intuitive for the spatial distribution relationships presented. Therefore, the marine-related industries are visualized by clustering, and the clustering visualization better presents the regional heat correlation of marine industries. As can be seen from Figure 9, the heat of the marine industry in Siming District and Tongan District is greater, which indicates the optimistic development of the marine economy in the region, while the rest of the region needs to make more efforts on economic transformation.

## 4. Conclusion

To address the problem that the existing recommendation algorithms based on the knowledge graph are limited to the integration of information of a single path neighborhood and ignore the combination and interaction between different neighborhoods of the knowledge graph, we propose and implement a recommendation ranking framework based on the breadth and depth of the knowledge graph, which achieves the construction of finite higher-order combination features of the triad of the knowledge graph from the breadth dimension and the integration of the path information of the multihop neighborhood of the knowledge graph from the depth dimension. The framework is based on the integration of multihop neighborhood path information of the knowledge graph, modeling the explicit and implicit interest preferences of users and effectively improve the accuracy of recommendation ranking results. This includes the legal and regulatory system of network intellectual property, the administrative enforcement system of network intellectual property, and the platform autonomy system of network intellectual property protection. Institutional guarantee strategy for the coordinated development of network ecology and industrial economy. The institution for the coordinated development of network ecology and the industrial economy is especially responsible for drawing up the plan for the coordinated development of network ecology and industrial economy, formulating policies for the coordinated development of network ecology and industrial economy, strengthening communication and cooperation among various departments, and so on. to promote the coordinated development of network ecology and industrial economy. Financial guarantee strategy, mainly including network infrastructure and network information technology research and development funds. This study refines the network ecology and industrial economy-coordinated development logo initially with the help of brainstorming method and combines the consistency test to delete and modify the initially screened logo to come up with the final network ecology and industrial economy-coordinated development logo.

## Data Availability

The data used to support the findings of this study are available from the corresponding author upon request.
